# Three-dimensional Versus Two-dimensional Laparoscopic Bariatric Surgery: A Systematic Review and Meta-analysis

**DOI:** 10.1007/s11695-024-07222-4

**Published:** 2024-04-17

**Authors:** Roberto Peltrini, Maria Danila Esposito, Daniela Pacella, Pietro Calabrese, Antonio Vitiello, Vincenzo Pilone

**Affiliations:** 1 Department of Public Health, University of Naples Federico II, Via Pansini 5, 80131 Naples, Italy; 2 Department of General Surgery, Transplantation and Gastroenterology, Federico II University Hospital, Via Pansini 5, 80131 Naples, Italy; 3 Department of Advanced Biomedical Sciences, University of Naples Federico II, Via Pansini 5, 80131 Naples, Italy

**Keywords:** 3D laparoscopy, 2D laparoscopy, Sleeve gastrectomy, Gastric By-pass

## Abstract

Three-dimensional (3D) laparoscopy has several advantages in gastrointestinal surgery. This systematic review determined whether similar benefits exist for bariatric surgical procedures by systematically searching the MEDLINE, Embase, and Scopus databases. Six studies including 629 patients who underwent 2D (386) and 3D (243) laparoscopic bariatric surgeries were selected. Operative time was significantly shorter in patients undergoing 3D laparoscopic gastric bypass (pooled standardized mean difference [SMD] 1.19, 95% confidence interval [CI] 2.22-0.15). Similarly, a shorter hospital stay was detected both during sleeve gastrectomy (SMD 0.42, 95% CI 0.70-0.13) and gastric bypass (SMD 0.39, 95% CI 0.64-0.14) with 3D laparoscopy. The study showed the potential benefit of 3D imaging in preventing intra- and postoperative complications. Despite the limited evidence, surgeons may benefit from 3D laparoscopy during bariatric surgery.

**Supplementary Information:**

The online version contains supplementary material available at 10.1007/s11695-024-07222-4.

## Introduction

The development of laparoscopic vision platforms has promoted technological innovations in minimally invasive surgeries [1]. Most surgeons routinely use two-dimensional (2D) laparoscopy, which provides unfavorable images in terms of depth perception and hand-eye coordination [2]. Three-dimensional (3D) laparoscopy has introduced a new perspective for minimally invasive surgical procedures in the field of general surgery. It overcomes the disadvantage of a traditional 2D laparoscopic system by improving depth perception and hand-eye coordination. These advantages are relevant during complex laparoscopic tasks such as tissue dissection and manipulation, suturing, and knotting [1]. In 2016, Cheng et al. conducted a systematic review and meta-analysis to investigate the advantages of 3D laparoscopy over 2D laparoscopy in different fields of surgery. This unequivocally demonstrated that the surgical duration of 3D laparoscopy was much shorter than that of the 2D technique and recommended 3D laparoscopy mainly for cholecystectomy and prostatectomy because a more stereoscopic visual perception facilitates tissue separation and vessel ligation [3]. Other studies have compared 2D and 3D laparoscopies in different fields of surgery, showing a decrease in operative time with 3D laparoscopy during cholecystectomy and transanal total mesorectal excision [1, 4, 5].

Similarly, the use of 3D laparoscopy has been investigated even during surgeries for pathological obesity. Therefore, the present study provides a systematic review of the literature that aims to assess whether 3D vision offers advantages, even during the surgical treatment of obesity.

## Materials and Methods

The Preferred Reporting Items for Systematic Reviews and Meta-Analysis (PRISMA) guidelines were followed in the database searches [6].

### Search and Study Selection

A systematic literature search was conducted to identify cohort trials that compared 2D and 3D laparoscopies for bariatric surgery. The MEDLINE (PubMed), Scopus, and Web of Science (WOS) databases were accessed and systematically searched on August 3, 2023 for relevant human clinical trials pertaining to the established topic. The following keywords were used: “3D,” “three dimensional,” “sleeve gastrectomy,” “vertical gastrectomy,” “mini bypass,” “Roux en-y gastric bypass,” “bariatric surgery,” “laparoscopy,” and “laparoscopic.” In this study, the following PICO model was used: patients undergoing 3D bariatric surgery (sleeve gastrectomy [SG] and/or gastric bypass [GB]) were compared with those undergoing 2D bariatric surgery with respect to operative time, length of hospital stay, intraoperative complications, and postoperative complications.

### Eligibility Criteria

All studies that met the eligibility criteria were evaluated by two independent reviewers (R.P. and M.D.E.) and any conflicts were resolved through discussion. A double-blind procedure was used to increase the precision of the extracted data. Studies that met the following criteria were included: comparative trials focusing on bariatric surgery and comparing 3D and 2D laparoscopic surgical procedures. A bibliographic review of the selected articles was performed as a secondary source of full-length articles. Full texts that were not available in English, review articles, case reports, conference papers, technical notes, and duplicate publications were excluded. The screening process was conducted using Rayyan (http://rayyan.qcri.org), with titles and abstracts screened before a full-text review.

### Data Extraction and Quality Assessment

Data were independently extracted and entered into Excel spreadsheets (Microsoft Inc., Redmond, Washington, USA). The following data were obtained from each study: first author, study design, study period, type of surgery, total number of participants, number of participants in each group, operative time, length of hospital stay, global complications, intraoperative complications, postoperative complications, and follow-up period. All the studies were assessed for methodological quality. For prospective, non-randomized, and retrospective studies, the Newcastle–Ottawa scale was used, with an overall score ranging from 0 to 9. The Newcastle–Ottawa quality assessment scale assigns a maximum of 9 points for the least risk of bias in three domains: (a) selection of study groups (4 points), (b) comparability of groups (2 points), and (c) ascertainment of exposure (3 points) for case-control studies [7]. The assignments were performed before the start of the study. For the randomized studies, a validated Jadad scale was used. The scale ranged from 0 to 5 and consisted of three items pertaining to descriptions of randomization, blinding, and an account of all patients [8].

### Statistical Analysis

Quantitative analysis was performed on the aggregate data from the selected studies. The standardized mean difference (SMD) was used to summarize the pooled difference between the means of the two groups (3D vs. 2D) for continuous variables. When mean and standard deviation (SD) were not available or reported but median and interquartile range were available, the Hozo method of converting median and range to mean and SD was used [9]. Where the SD was not reported but the mean and p-value were available, the estimate of the pooled SD was derived from the test statistical formula, assuming equal variances. The random-effects model was used for the pooled analysis when data from all three studies were available. When data from only two studies were available, a common-effect model was applied. Heterogeneity was assessed using both I2 statistic and Kendall's Τ. The studies were considered highly heterogeneous when p < 0.05. All analyses were conducted using the R statistical software version 4.3.1. Common and random effects models were applied using the meta-package for R.

## Results

**Fig. 1:**
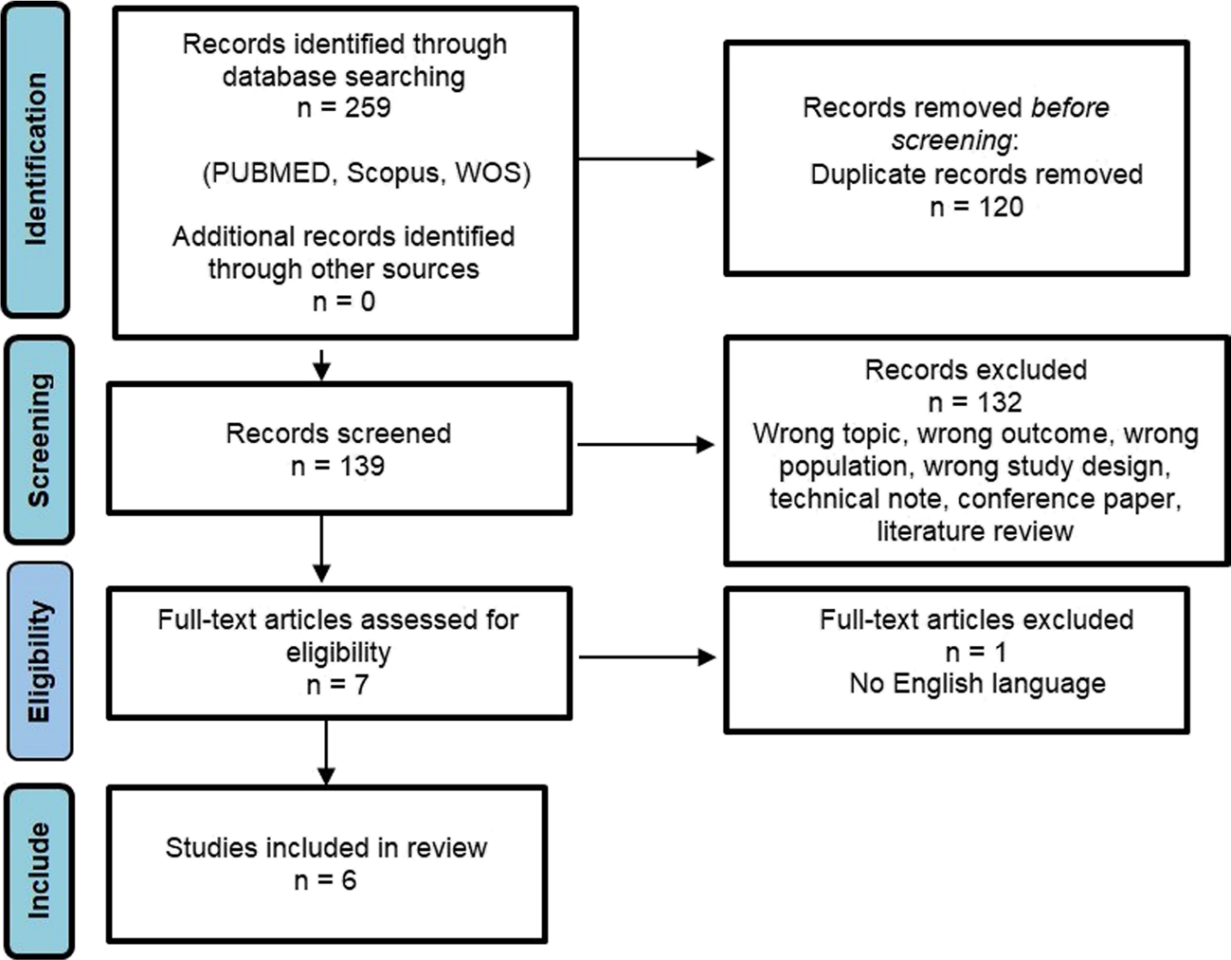
PRISMA Flow Diagram

The PRISMA flowchart is shown in Fig. 1. A total of 259 studies were identified based on the search strategy. Duplicate publications were also excluded (n=120). After screening the title and abstract, 132 papers were excluded, and the full texts of seven studies were assessed for eligibility. Another study was excluded because it was not conducted in English. Finally, only six studies [2, 10–14] were included in the present meta-analysis based on the aforementioned inclusion criteria. These were all single-center studies including four prospective [10–13] and two retrospective [2, 14] studies. A total of 629 patients who underwent 3D or 2D laparoscopic bariatric surgery were included in the meta-analysis. Among them, 386 and 243 patients underwent 2D and 3D laparoscopic bariatric surgeries, respectively. Table 1 presents the characteristics of the included studies. In addition, baseline patients’ characteristics were reported in Table S1. The distribution by gender, BMI and comorbidities was similar in both groups with no significant difference in all studies except for the cohort of patients analysed by Padin et al. [14] where there was a difference in BMI (44.59 ± 6.68 in 3D and 46.61 ± 6.48 in 2D group; p=0.008).

**Table 1:** Details of the included studies

	Study design	Study period	Type of Surgery	Group 2D	Group 3D	Follow up (months)	Qualitative assessment
Curro et al. [10]	RCT	/	SG	10	10	N/D	3/5*
Padin et al. [14]	Retrospective Study	2013 - 2016	SG	92	44	1	7/9**
Martínez-Ubieto et al. [12]	Prospective Study	2013 - 2015	SG	37	41	24	7/9**
Curro et al. [10]	RCT	/	OAGB	10	10	N/D	3/5*
Padin et al. [14]	Retrospective Study	2013 - 2016	RYGB	116	60	1	7/9**
Rojano Rodrìguez et al. [13]	RCT	/	RYGB	18	20	N/D	3/5*
Mongelli et al. [2]	Retrospective Study	2014 - 2018	RYGB	78	33	1	9/9**
Gabrielli et al. [11]	Prospective Study	2018	RYGB	25	25	1	9/9**

*RCT* Randomized Controlled Trial, *SG* Sleeve Gastrectomy, *OAGB* One Anastomosis Gastric Bypass, *RYGB* Roux en Y Gastric Bypass, *Jadad Scale – ** Newcastle Ottawa Scale

### Operative Time

**Fig. 2:**
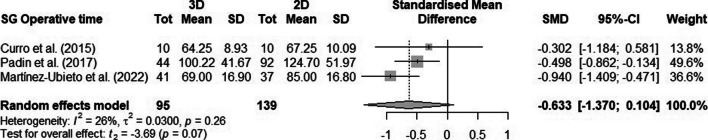
Forest plot of comparison of operative time for Sleeve Gastrectomy

**Fig. 3:**
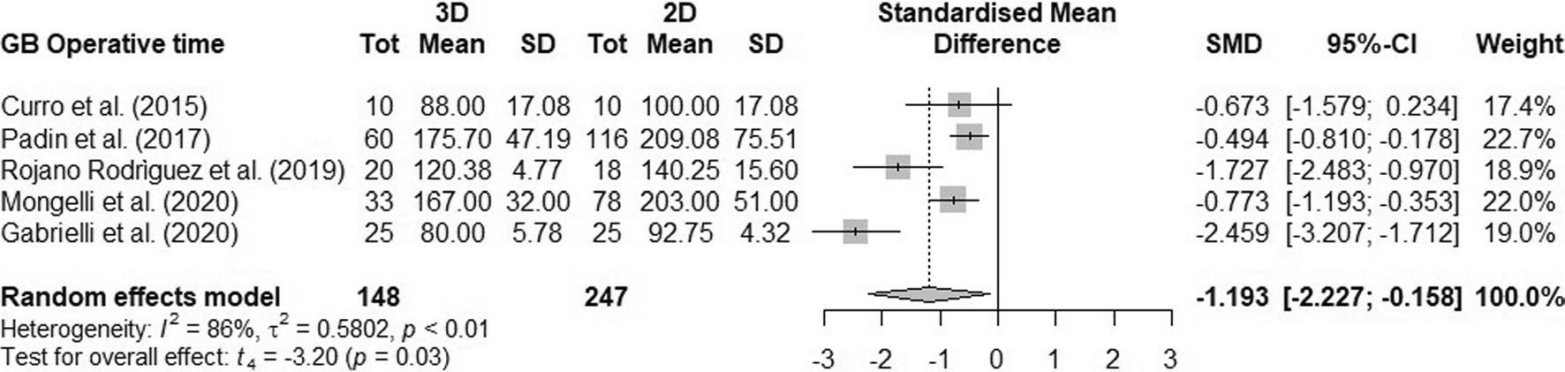
Forest plot of comparison of operative time for Gastric Bypass

In the SG group [10, 11, 13], the pooled SMD was 0.63 (95% confidence interval [CI] 1.37-0.10). Low heterogeneity was observed (I2=26%, p=0.26) (Fig. 2). Concerning the GB group [2, 10, 11, 13, 14], the pooled SMD was 1.19 (95% CI 2.22-0.15). High heterogeneity was observed (I2 = 86%, P < 0.01) (Fig. 3).

### Length of Hospital Stay

**Fig. 4:**
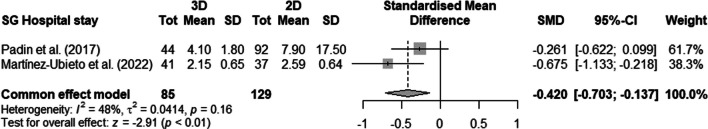
Forest plot of comparison of Hospital Stay for Sleeve Gastrectomy

**Fig. 5:**
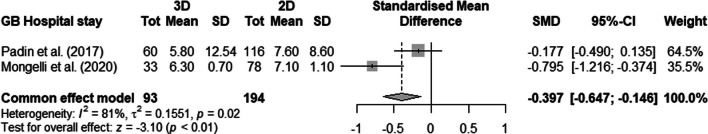
Forest plot of comparison of Hospital Stay for Gastric Bypass

Considering the length of stay for the SG group [12, 14], the pooled SMD obtained was 0.42 (95% CI 0.70-0.13). Moderate heterogeneity was observed (I2=48%, p=0.16) (Fig. 4). In the GB group [2, 14], the pooled SMD was 0.39 (95% CI 0.64-0.14). High heterogeneity was observed (I2=81%, p < 0.01) (Fig. 5).

### Intra- and Postoperative Complications

A qualitative synthesis of the overall postoperative complications, such as fistulas, stenosis, and bleeding, is shown (Table 2).

**Table 2:** Outcomes after 3D and 2D laparoscopic bariatric surgery

References	Type of Surgery	Overall complications 2D Group n/N (%)	Overall complications 3D Group n/N (%)	Operative time 2D 3D (minutes)		Hospital Stay 2D 3D (days)		Surgeon experience	Previous 3D system Surgeon experience
Curro et al. [10]	SG	0/10 (0)	0/10 (0)	72 [45 - 80]	68 [45 - 76]	N/D	N/D	One experienced surgeon (around 350 bariatric procedures)	Two SG
Padin et al. [14]	SG	4/92 (4.3) 2 Fistula 2 Pneumonia	0/92 (0)	124.7 ± 51.97	100.22 ± 41.67	7.9 ± 17.5	4.1 ± 1.8	One experienced surgeon (more than 250 bariatric procedures); three surgeons with less than 50 bariatric procedures	None
Martínez-Ubieto et al. [12]	SG	8/37 (21.6) 3 Atelectasis 1 Sleeve Stenosis 3 Fistula 1 Peritonitis	6/41 (14.6) 1 Atelectasis 1 Malnutrition 1 Guillan-Barre Syndrome 2 Stenosis 1 Fistula	85 ± 16.8	69 ± 16.9	2.59 ± 0.64	2.15 ± 0.65	Surgical team with extensive experience in advanced laparoscopic surgery	N/D
Curro et al. [10]	OAGB	0/10 (0)	0/10 (0)	100	88	N/D	N/D	One experienced surgeon (around 350 bariatric procedures)	Two OAGB
Padin et al. [14]	GB	8/116 (6.8) 4 Fistula 1 Hemoperitoneum 2 Upper GI bleeding 1 Obstruction	1/60 (1.6) 1 Upper GI bleeding	209.08 ± 75.51	175.7 ± 47.19	7.6 ± 8.6	5.8 ± 12.54	One experienced surgeon (more than 250 bariatric procedures); three surgeons with less than 50 bariatric procedures	None
Rojano Rodrìguez et al. [13]	GB	0/18 (0)	0/20 (0)	136.5 [117-171]	120 [112.5-129]	N/D	N/D	N/D	N/D
Mongelli et al. [2]	RYGB	7/78 (8.9) 1 Inadequate dimension of the gastric pouch 1 tension on the gastrojejunal anastomosis 1 liver bleeding 1 bowel ischemia 1 anastomotic leakage 2 postoperative bleeding	2/33 (6.0) 1 iatrogenic ileal injury 1 bowel ischemia 1 anastomotic leakage 2 postoperative bleeding	203 ± 51	167 ± 32	7.1 ± 1.1	6.3 ± 0.7	Experienced bariatric surgeons	N/D
Gabrielli et al. [11]	RYGB	0/25 (0)	1/25 (4.0) 1 bleeding	93 [85-100]	80 [70-90]	N/D	N/D	Surgical team with more than 500 laparoscopic procedures using 2D system	Surgical team with more than 100 laparoscopic procedures using 3D system

*SG* Sleeve Gastrectomy, *OAGB* One Anastomosis Gastric Bypass, *RYGB* Roux en Y Gastric Bypass

Three studies [10, 11, 13] that compared laparoscopic procedures performed using 3D and 2D video systems reported neither intra- nor postoperative complications in either group.

In other studies [2, 12], no statistically significant values were observed for global complications, except for that by Padin et al., who analyzed the occurrence of complications based on the surgeon's experience at the beginning of the study. In this study, complications were recorded in the group of patients who were operated on by novice surgeons. However, when comparing the percentage of complications between the 2D and 3D cohorts, a reduction in the number of events from 10.2% to 1.8% was observed.

No significant differences were noted in terms of bleeding or stenosis between the 3D and 2D groups. Regarding the incidence of postoperative fistulas, Padin et al. reported a statistically significant p-value in a comparison between 3D and 2D cohorts of patients operated on by novice surgeons, with better results for the use of the 3D video system. Martinez et al. reported fistulas as the most frequent complication, occurring at a rate of 8.1% in the 2D group and 2.43% in the 3D group. Additionally, they documented a case of peritonitis, classified as the most severe complication (Clavien–Dindo grade IVa [15]), which occurred in a single patient undergoing 2D SG and was treated with reoperation.

## Discussion

This meta-analysis is the first to investigate the potential advantages of 3D over 2D laparoscopy for bariatric surgery. In our study, we included trials that compared two video systems for SG [10, 12, 14] and GB [2, 10, 11, 13, 14] procedures. 3D laparoscopy resulted in a significant reduction in the operative time and length of hospital stay among patients undergoing bariatric surgery.

The duration of the surgical procedure influences the risk of rhabdomyolysis, which increases with prolonged immobilization [16]. A shorter duration of surgical procedures decreases the exposure of patients with obesity to anesthetics and the rate of pulmonary complications, including pulmonary embolism secondary to deep vein thrombosis. In fact, prolonged surgery duration and immobilization are independent predictors of postoperative lung diseases, such as atelectasis, pneumonia, pulmonary embolism, and respiratory failure [17, 18]. Prolonged postoperative hospital stay increases hospital costs and the risk of infections and is a predictor of readmission in patients undergoing bariatric surgery [19–21].

These results are consistent with those of other previously published meta-analyses or trials investigating the efficacy of 3D and 2D laparoscopies. Cheng et al. [3] observed a shorter duration for surgical procedures in patients undergoing cholecystectomy, prostatectomy, or digestive surgery using a 3D video system. In addition, a randomized controlled trial comparing colon cancer resection performed using 3D and 2D laparoscopies found advantages only in terms of the duration of the surgical procedure in the 3D group, with no observed differences in the length of postoperative hospital stay between the two groups [22].

In the present study, the operative time was significantly shorter in patients undergoing one anastomosis gastric bypass [10] and RYGB [2, 11, 13, 14] with 3D laparoscopy, while there was no statistical difference between the two video systems in patients undergoing SG [10, 14]. This result can be attributed to the fact that the use of 3D may be of little importance in simple tasks, but can increase task efficiency, particularly during the execution of difficult surgical tasks [23], such as suturing and knotting [10, 24]. Indeed, during Roux-en-Y gastric bypass surgery, hand-sewing gastrojejunal anastomosis requires great skill and involves complex interactions between planes [13].

Prolonged recovery can increase the risk of serious postoperative complications, such as hospital-acquired infections [25]. A significant reduction in postoperative hospitalization time was observed in patients undergoing GB. Two studies were evaluated in this setting. Mongelli et al. [2] found that operative time, longer in 2D group, was the only factor independently associated with a prolonged hospital stay. In the study of Padin et al. [14] the high complication rate (4.3% vs 0%) was perhaps the basis for the longer hospital stay of these patients. However, the statistical heterogeneity was high (*I*^2^ = 81%, *P* = 0.02) and it cannot be excluded that our findings were influenced by inherent bias and this constitutes a relevant limitation of the study.

The follow up was reported in four of the six included studies. Complications were observed during recovery and within 30 days of surgery in three studies. Only Martínez-Ubieto et al [12] reported a follow-up of 24 months. As follow up differs among the studies with limited description of the identification of complications, this is considered a further limitation of the study.

Owing to the limited number of complications in the included studies, a narrative synthesis of the results was performed. A high overall complication rate was reported by Martínez-Ubieto et al. [12] in both 2D and 3D groups (22% and 15%, respectively). This is probably due to the included non-surgical and Clavien-Dindo grade I and II complications and a longer follow up. Padin et al. [14] reported a reduction in complications in the 3D cohort compared with the 2D cohort, especially in patients operated on by novice surgeons. The experience of the surgeons performing the procedures may have influenced the study results. Laparoscopic surgeons require extensive experience to overcome the lack of depth perception. However, with experience, the operator becomes accustomed to 2D vision. Experienced laparoscopic surgeons do not require a stereoscopic view to perform simple tasks such as dividing the greater curvature ligaments or constructing the gastric tube during SG using linear staplers [10]. Furthermore, 3D laparoscopy appears to help novice surgeons in reducing complications [14]. Although surgeons are largely skilled in laparoscopic bariatric procedures, there was a limited information on previous experience with 3D system. The surgical team performed more than 100 laparoscopic procedures using 3D laparoscopy in the study of Gabrielli et al. [11]. In contrast, very few or no procedures have been performed in other studies [10, 14]. Despite the surgical team/surgeon was the same for both groups (2D vs 3D) in each included study, surgical background may have a not negligible impact on our findings.

3D laparoscopy may have some disadvantages. The stereoscopic effect may cause headache, nausea and eye strain [26–28]. The operating team also must wear polarized glasses to view the screen in 3D with potential inconveniences. Moreover, the benefits of 3D vision such as better depth perception, image quality and spatial orientation may have a limited impact for experienced and skilled laparoscopic surgeons.

## Conclusion

The use of 3D video systems in bariatric surgery appears to significantly decrease operative time during GB and recovery in all surgical procedures. Therefore, adoption of 3D vision may be advantageous in this context. However, since the evidence is still limited due to the small number of studies, firm conclusions cannot be drawn, and additional high-quality studies are warranted.

### Supplementary Information

ESM 1 Table S1 Baseline patients’ characteristics. BMI body mass index, DM diabetes mellitus, AHT arterial hypertension, OSAS obstructive sleep apnea syndrome. (DOCX 90 kb)
