# Supplementary figures and images for: Three-dimensional Versus Two-dimensional Laparoscopic Bariatric Surgery: A Systematic Review and Meta-analysis
Source: Obes Surg. 2024 Apr 17;34(6):2177–85. doi: 10.1007/s11695-024-07222-4 (PMC11127895; doi:10.1007/s11695-024-07222-4)

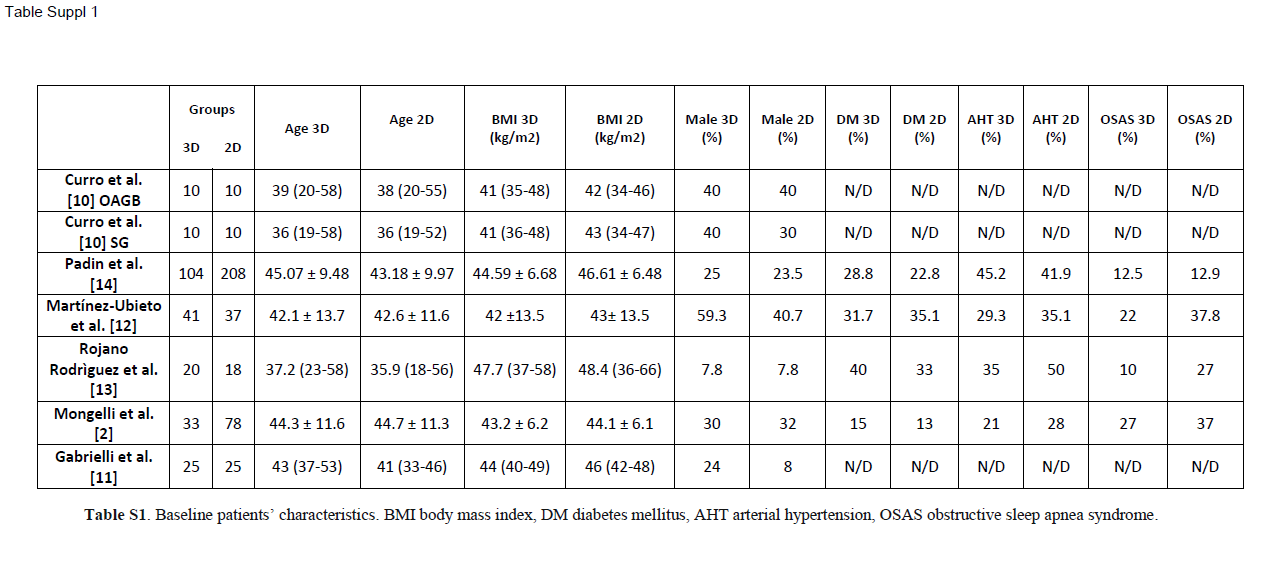

Supplement: Supplementary file 1 — Table S1 Baseline patients’ characteristics. BMI body mass index, DM diabetes mellitus, AHT arterial hypertension, OSAS obstructive sleep apnea syndrome. (DOCX 90 kb) [file 11695_2024_7222_MOESM1_ESM.docx]
